# Sexual health after childbirth: a qualitative study of women’s experiences

**DOI:** 10.1186/s12978-026-02458-y

**Published:** 2026-09-25

**Authors:** Lisa Goldkuhl, Linda Åkeflo, Elin Wallner, Helen Elden

**Affiliations:** 1 Institute of Health and Care Sciences, Sahlgrenska Academy, University of Gothenburg, Box 457, Gothenburg, 405 30 Sweden; 2 Department of Obstetrics and Gynaecology, Sahlgrenska University Hospital, Region Västra Götaland, Gothenburg, Sweden; 3 Department of Oncology, Sahlgrenska University Hospital, Gothenburg, Sweden; 4 Institute of Clinical Sciences, Sahlgrenska Academy, University of Gothenburg, Gothenburg, Sweden; 5 Centre for Sexology and Sexuality Studies, Malmö University, Malmö, Sweden; 6 Department of Culture and Media Studies, Umeå University, Umeå, Sweden

**Keywords:** Sexual health, Women’s health, Postpartum, Qualitative research, Thematic analysis, Queer phenomenology

## Abstract

**Background:**

Sexual health is a vital component of overall well-being but receives limited attention in postpartum care. This study aimed to explore how pregnancy and childbirth shape women’s experiences of sexual health, and to identify how care and support can be tailored to promote sexual well-being after childbirth.

**Methods:**

We conducted an inductive qualitative study based on individual interviews with twenty women who had given birth in Sweden within the previous two years. Data were analysed using Braun and Clarke’s reflexive thematic analysis. The resulting themes were subsequently interpreted through Sara Ahmed’s queer phenomenology.

**Findings:**

One overarching theme, *Reorienting to sexual health after childbirth*, described how participants viewed their sexual health as a process of adapting to physical, emotional, and relational changes rather than returning to pre-pregnancy experiences. Four sub-themes were identified: (1) *Reviving desire in a changed body* (2), *Living with the impact of the childbirth experience* (3), *Balancing parental and sexual identity*, and (4) *Navigating postpartum sexual health without support.* Participants generally perceived healthcare support after childbirth as inadequate, as it reinforced this narrow framing through a focus on contraceptive counselling and readiness for intercourse rather than sexual health more broadly. Drawing on queer phenomenology, healthcare support appeared to be shaped by heteronormative assumptions, while women re-oriented their sexual health after childbirth both within and beyond heteronormative sexual norms.

**Conclusion:**

Postpartum sexual health should be addressed as a broader aspect of well-being rather than being reduced to contraceptive needs or the resumption of intercourse. Care should be individualised, acknowledge variations in desire and intimacy, and ensure that persistent symptoms and concerns receive appropriate assessment and support.

**Supplementary Information:**

The online version contains supplementary material available at https://doi.org/10.1186/s12978-026-02458-y.

## Abstract (plain language summary)

Sexual health is an important part of well-being, but it is often not talked about in routine care after childbirth. In this study, we wanted to understand how women experience sexual health in the months and years after pregnancy and childbirth, and what kind of support they feel is missing. We interviewed 20 women in Sweden who had given birth within the previous two years and analysed their stories to identify shared patterns.

Women described sexual health after childbirth as a process of “finding a new normal” rather than returning to how things were before pregnancy. Many needed time to adjust to bodily changes, tiredness, pain, and changes in how they felt about their bodies. Childbirth experiences could also shape sexual well-being afterwards. Some women felt stronger and more confident, while others felt fear, distress, or disconnection.

Parenthood also changed everyday life and relationships. Some women found new ways of being close with a partner, while others struggled to balance caring for a baby with feeling like a partner and a sexual person. Most women said they received little or no support from healthcare services about sexual health after childbirth. Conversations were often limited to contraception and whether vaginal intercourse had resumed, leaving many questions and concerns unanswered.

Our findings suggest postpartum care should include more open, individualised conversations about sexual health, including intimacy, pleasure, comfort, and relationship needs and not only physical recovery and contraception.

**Supplementary Information:**

The online version contains supplementary material available at https://doi.org/10.1186/s12978-026-02458-y.

## Background

Pregnancy and childbirth bring significant physical, emotional, and social changes that affect many aspects of a woman’s life. Sexual health is often affected, with shifts occurring both on an individual level and within intimate relationships [1]. While sexual health involves a range of experiences, much of the existing literature has focused on defining postpartum sexual health in terms of sexual function or dysfunction [2–4], with the timing of vaginal intercourse resumption often used as the primary outcome [5]. This approach limits the focus to observable and heteronormative behaviours instead of acknowledging the complexity of lived sexual experiences. Such narrowing reflects a trend where sexual health is often conflated with risk prevention and reproductive function [6].

According to the Guttmacher-Lancet Commission [7], sexual and reproductive health and rights represent more than the absence of disease or dysfunction. They also include the right to make informed decisions about one’s body, access services that support those decisions, and experience sexuality with safety, trust, and mutual respect. Linear models of sexual response describe a sequential progression from arousal to orgasm [8]. Alternative approaches recognise that sexual desire is also shaped by emotional intimacy, relationship factors, and context, and may emerge in response to rather than precede sexual activity [9]. This broader perspective is particularly relevant after childbirth, when intimacy, body image, and sexual identity may change [2].

The postpartum period, defined by the World Health Organization (WHO) as a critical yet often neglected phase of maternal care, marks a time of physical recovery and emotional adjustment [10]. Although traditionally defined as a six-to-eight week phase, recovery after childbirth is increasingly understood to extend for a year or more [11, 12]. During this time, many women experience ongoing physical and psychological challenges, such as fatigue, pain, decreased sexual desire due to hormonal changes, sleep disturbances, anxiety, and body image concerns [1, 13]. Support from healthcare professionals during this period is essential, as inadequate care may increase the risk of breastfeeding difficulties, postpartum depression, and impaired mother-infant bonding [10, 13].

Qualitative studies have broadened understanding of sexual health after childbirth by describing changes in embodiment and sexual identity, intimacy and relationships, readiness for sexual activity, and strategies for adapting to a changed sexual life [14–17]. One recent study also described how women with persistent health problems after severe perineal trauma experienced their symptoms as being normalised or insufficiently acknowledged in healthcare encounters [18]. Despite these contributions, less is known about how women understand sexual health as a whole and how bodily and relational changes intersect with social norms, identity, and healthcare experiences. This study therefore aimed to explore how pregnancy and childbirth shape women’s lived experiences of sexual health, and to identify how care and support can be tailored to promote sexual well-being after childbirth.

## Methods

A qualitative study with an inductive design was conducted based on semi-structured interviews with twenty women who had given birth in Sweden during the years 2022 to 2024. The study procedures and reporting of the manuscript have followed the Standards for Reporting Qualitative Research (SRQR; Supplementary material 1) [19].

### Setting

Postpartum care in Sweden is an integral part of the national maternal health programme and aims to support physical and emotional recovery after childbirth. On average, the hospital stay following a vaginal birth is two days. In some regions, early discharge is practised if no complications have arisen where families return home within 6 to 12 h, with planned follow-up in outpatient care. A routine postpartum visit, typically conducted by a midwife, is offered within six to twelve weeks after the birth. This commonly includes discussions about the childbirth experience, contraceptive counselling, and assessments of the woman’s physical and mental health. In some Swedish regions, one digital follow-up appointment is offered within the first two weeks postpartum in addition to the physical visit [20].

### Recruitment and participants

Participants were recruited through purposive sampling to seek variation in age, geographic location in Sweden, parity, country of birth, educational background, and birth outcomes (e.g., mode of birth, straightforward or with complications, positive or negative birth experiences). Inclusion criteria were age between 18 and 45 years, birth of at least one live infant between six weeks and two years prior to the interview, and ability to communicate in Swedish or English.

Study information was distributed by email to managers at child health centres and midwifery clinics providing postpartum care across Sweden, with a request to display recruitment posters in waiting areas. The announcement was also shared through networks connected to the Public Health Agency of Sweden and the research team. Interested women contacted the first author directly and were given additional information and the opportunity to ask questions. If inclusion criteria were met and the person agreed to participate, the interview was scheduled at a time and location of their choice.

To reach participants born outside Sweden, recruitment included an in-person visit to a midwifery clinic, where midwives subsequently informed eligible individuals about the study. Additional outreach was conducted through Facebook groups for new parents who identified with a gender other than woman and/or a non-heterosexual orientation. Despite these efforts, only cisgender heterosexual women enrolled. In total, twenty participants were included who had all given birth in Sweden. They resided in six different regions, including rural areas, small towns, and cities. Participant characteristics are summarised in Table 1.

**Table 1:** Participant characteristics (*n* = 20)

Age	
Range (mean)	25–43 (33)
Parity	
Primiparous	11
Parous ^a^	9
Months since last childbirth (mean)	2–23 (13)
Mode of birth	
Spontaneous vaginal	11
Caesarean	9 ^b^
Exclusively breastfed for 6 months	17
Reported postpartum complications ^c^	6
Relationship status	
Married ^d^	11
Cohabiting ^d^	8
Single	1
Country of birth	
Sweden	16
Other European country	2
African country	2
Highest level of education	
Primary school	1
Upper secondary school	3
University or college	16

^a^Had 2–6 children

^b^Two of these women had previously given birth vaginally

^c^Including severe bleeding, slow-healing surgical wounds, or incorrectly repaired perineal tears

^d^All partnered participants were in relationships with men

### Data collection

Data were collected between June and November 2024 by two of the authors (LG and LÅ), both experienced in qualitative methodology. A semi-structured interview guide was developed to explore participants’ experiences of sexual health after childbirth (Supplementary material 2). The opening questions were: “What does sexual health mean to you?” and “How has your sexual health changed since pregnancy and childbirth?” Follow-up questions were asked in response to participants’ answers to further explore their perspectives. The guide was pilot-tested with the first participant and subsequently revised to clarify questions regarding desired support from healthcare services.

Eighteen interviews were held via video calls and two by telephone. Nineteen interviews were conducted in Swedish and one in English. Interviews ranged from 35 to 65 min in length, were audio-recorded, and transcribed verbatim. Data collection ended after 20 interviews when the dataset was considered sufficiently rich, diverse, and relevant to support a meaningful interpretation of patterns in relation to the study aim [21].

### Data analysis

The transcribed interviews were analysed using Reflexive Thematic Analysis (RTA) as described by Braun and Clarke [21] to identify key themes across the data. The analysis began with repeated readings of the transcripts to establish familiarity, followed by systematic coding to capture meaningful content. Coding and data organisation were supported by NVivo software (version 14).

LG and LÅ conducted coding independently to deepen the interpretation and promote critical reflection, as opposed to ensuring inter-coder reliability, consistent with the RTA approach [21]. Preliminary interpretations were then discussed jointly to identify potential themes based on recurring patterns and meanings. These themes were further reviewed and refined through iterative discussions within the research group.

RTA recognises the contextual and interpretive nature of qualitative analysis, and the researchers’ perspectives were viewed as a resource rather than a bias. The research team included two midwives experienced in intrapartum and postpartum care, a registered nurse with expertise in sexual health after gynaecological cancer, and an ethnologist specialised in phenomenological and norm critical perspectives on women’s healthcare encounters. This multidisciplinary group provided a combination of clinical knowledge, subject expertise, and critical distance. Reflexivity was operationalised through critical discussion of preliminary themes within the research team, including consideration of how the researchers’ clinical, disciplinary, and theoretical backgrounds could shape interpretations. Alternative interpretations were actively considered during the refinement of the themes.

### Theoretical perspective

After the initial themes had been developed through RTA, Sara Ahmed’s queer phenomenology [22] was applied as a theoretical perspective to deepen their interpretation and further refine the themes. This perspective helped us examine variations and patterns in women’s lived experiences of sexual health after childbirth and how these experiences were shaped by cultural and normative expectations. Queer phenomenology has previously been applied to explore embodiment, sexuality, heteronormativity, and postpartum care [23–26]. It examines how bodies are oriented in time and space through social norms, cultural expectations, and institutional structures. According to Ahmed [22], orientations are habitual and shape which experiences become possible or recognised. Following normative “lines,” such as heterosexual coupledom and motherhood, may allow some bodies to feel at ease, whereas deviation from these expectations may lead to disorientation or exclusion.

Regarding sexual health after childbirth, this perspective helps explain how social norms surrounding motherhood, recovery, and sexuality shape how individuals relate to their bodies and sense of self. It provides understanding of how some may feel limited, stigmatised, or unsupported in healthcare settings that fail to recognise diverse identities and experiences [22, 26].

## Results

**Fig. 1:**
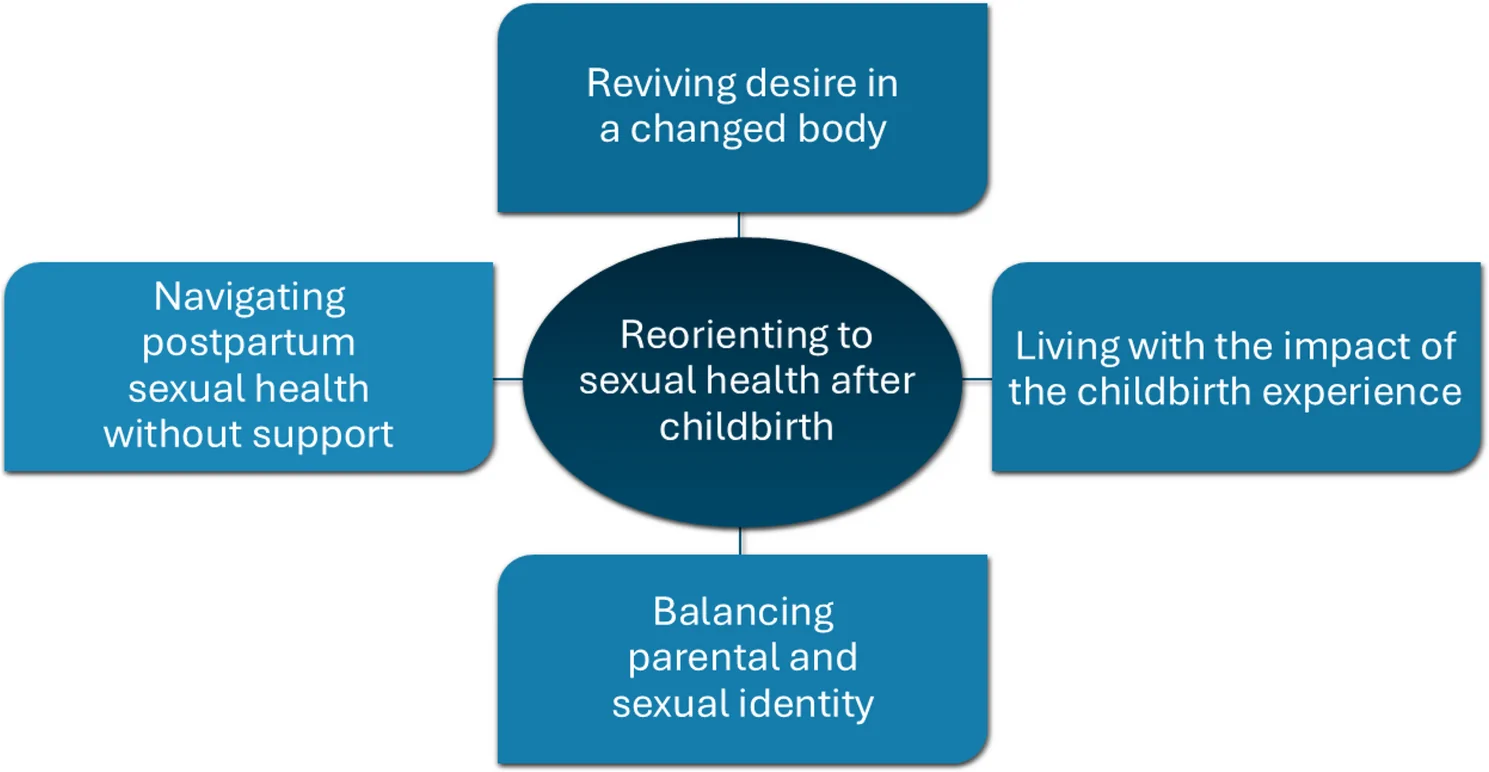
Themes illustrating how pregnancy and childbirth shaped the women’ sexual health

The analysis identified one overarching theme: *Reorienting to sexual health after childbirth* and four subthemes (Fig. 1). The overarching theme reflects participants’ experiences of sexual health after childbirth as an ongoing process of adjustment and redefinition rather than as a return to pre-pregnancy experiences. This reorientation involved coming to terms with physical and emotional changes, while seeking new ways to relate to one’s body, identity, and sexuality within a new life situation. Participants commonly described sexual health as multidimensional, involving a healthy relationship with their own body, a sense of autonomy, emotional and physical desire, and shared intimacy with a partner.

Sexual health remained an important part of the participants’ well-being after childbirth, even though sexual activity often became less central. Their experiences varied widely, and there was no single way to “return” to sexual health. Instead, it was understood as a complex and personal process that evolved over time. For many, this process took shape despite being unaddressed within a healthcare system that rarely considered sexuality beyond contraception and the expectation of resuming vaginal intercourse with a partner.

### Reviving desire in a changed body

After giving birth, many participants felt that their bodies had changed and no longer felt familiar, regardless of whether or not they had experienced major complications. They described a need to reconnect with their bodies and rethink the expectations related to sexuality and relationships. These bodily changes were often experienced as long-lasting, and included vaginal dryness, leaking breasts, altered appearance, and changed bodily sensation, such as numbness, discomfort, or pain.

“Overall, it just feels like I have less sensation. I’m not sure if it’s because some nerves didn’t heal properly, or if it’s the scar tissue from the stitches. But either I feel nothing at all, it feels unpleasant, or it hurts.” (Participant 5, 22 months after vaginal birth).

Participants differed in how they processed and came to accept these bodily changes, with some describing estrangement from their body and sexuality that affected their sexual well-being. This estrangement was closely linked to a reduced or absent sense of sexual desire, which was experienced as emerging from bodily discomfort rather than emotional disconnection. A decline in sexual desire during the postpartum period was common. “Returning to sex” was understood as resuming vaginal intercourse with a partner, while other expressions of sexuality, such as masturbation or non-penetrative intimacy, were rarely mentioned in the interviews. The timing for resuming sexual activity after childbirth varied widely, from a few weeks to more than a year, and was influenced by emotional and physical readiness, cultural norms, and childbirth experiences. While some had internalised expectations about when sex “should” resume, others emphasised that the return to sexual activity should be guided by desire.

“If your mind and body aren’t in sync, that’s when it becomes difficult. […] After my other children, I didn’t even wait the full 40 days, it felt easy. But this time, my body is telling me to wait. It’s my body that decides and the will to resume that guides it.” (Participant 20, 2 months after childbirth).

Participants articulated both internal and external expectations around when and how to resume sex. Since vaginal intercourse was often framed as the normative endpoint, those who were not ready or preferred other forms of intimacy sometimes felt inadequate. Many wished for the space to pause sexual activity without feeling guilt or pressure while navigating a shifting sexual identity and a sense of disconnection from their former selves.

“Sometimes I feel like I’m not a sexual person anymore and that makes me a bit sad, because it’s fun, you know? But then I try to be kind to myself and think: we’re talking about a year or two in a whole lifetime. It’s okay to have other things on your mind.” (Participant 2, 22 months after childbirth).

Fear of pain during sexual activity was common and could contribute to anxiety, tension, and lowered arousal, which created a vicious cycle that seemed to make sexual activity more difficult to resume. Avoidance could further strengthen this pattern, particularly when sexual experiences had been negative. When intimacy was reinitiated in a positive way, it sometimes helped revive desire. Postpartum changes also prompted reflection on emotional connection and relationship dynamics, opening space to redefine intimacy and explore new ways of connecting.

“We’re working on gratitude and lowering the bar. It’s the journey that matters. Both of us don’t have to climax — no one has to. There doesn’t need to be penetration. Nothing really has to happen. Just closeness. That’s the focus now: to feel.” (Participant 15, 20 months after childbirth).

### Living with the impact of the childbirth experience

The childbirth experience influenced participants’ self-image, including their sense of identity and perceptions of their body’s capacity and worth. These changes, in turn, affected their sexual health. For some, childbirth had strengthened their connection to their own body. Carrying and giving birth to a child gave rise to a sense of pride, gratitude, and physical confidence. Scars and stretch marks were viewed as signs of strength and motherhood. This shift in perspective allowed participants to feel more at ease during intimacy.

“My body looks different, and I think I would have struggled with that before I had children. But now I’m proud of the loose skin on my belly and the fold over my C-section scar. And my breasts have changed too; I don’t think they’re as nice anymore, but I’m proud of what my body has been through. I’ve also stopped caring what it looks like during sex. I think I’ve become a bit braver.” (Participant 13, 23 months after childbirth).

Motherhood shifted the focus from pleasing others to attending to one’s own needs and desires. Giving birth led to a stronger sense that the body deserved care and respect, both from oneself and others. This was particularly evident among participants with prior negative sexual experiences.

“I don’t want to expose myself to harm or anything negative, pain or anything else, especially not sexually, because I want my daughter to have a mother who feels good about her body in every way.” (Participant 6, 11 months after childbirth).

In contrast, difficult or traumatic births deeply affected participants’ sense of self and sexual identity. The physical and emotional consequences of childbirth could create a sense of disconnection from the body. Scars, pelvic injuries, and medical interventions, such as repeated vaginal examinations during labour, left lasting impressions on how participants perceived their bodies. Their bodies could feel foreign or even disturbing as previous pleasure was replaced by discomfort, shame, or fear. One woman described feeling her body had failed her, leaving her with a sense of inadequacy. Such experiences challenged deeply held ideas about femininity and motherhood, and made intimacy feel out of reach.

“For me, the trauma felt like failure. I had failed as a woman, and that took the joy and desire out of intimacy. It gave me this sense of being unworthy, like I wasn’t enough, like I had nothing to give. My body couldn’t do what I believed defined womanhood. And that made it very difficult to be open to that kind of intimacy.” (Participant 10, 17 months after childbirth).

### Balancing parental and sexual identity

Parenthood reshaped identity and influenced participants’ sexual well-being. The maternal role often took precedence, while being a partner or a sexual individual receded into the background. Struggling with the tension and conflicting roles of societal expectations to be both devoted mothers and sexually available partners led to frustration and internal conflict.

“It’s these expectations of how women are supposed to be… you’re supposed to be a mum, but also a sexual partner. And I find it really hard to combine the two in my head. I struggle with the question: can I be a sexual person and also be a good mum? It’s like it has to be either-or, but at the same time both are expected.” (Participant 2, 22 months after childbirth).

Many shifted from viewing their bodies as sources of pleasure to regarding them as instruments of care. Breasts, for example, were no longer primarily associated with sexuality, but with breastfeeding and nurturing. This shift could distance participants from their sexual identity, as the body felt less like one’s own and more in the service of the child.

“My body belongs to my daughter now. I don’t feel like I need to have sex. It might sound silly, but if you think about it in an evolutionary way, my body is made to have children. After having my first and second, it feels like I’ve done my part.” (Participant 17, 6 months after childbirth).

The constant demands of caring for a baby also made it difficult to relax and be present in moments of intimacy. Participants described being physically and mentally “on alert,” even when the child was asleep. Desire, once spontaneous, now required conscious effort. For those who had gone long periods without sexual activity, or for whom sex had been tied to becoming pregnant or meeting a partner’s needs, reconnecting with their own sexuality was challenging. Some expressed a need to reclaim their bodies for themselves, free from expectations and external demands.

When experiencing sexual identity as altered or diminished, some described themselves as temporarily asexual. The pressures of daily life left little room for personal needs. Intimacy could feel like yet another task, particularly when unequal division of parenting responsibilities led to feelings of resentment.

“You kind of lose each other more now. It’s hard to focus on each other and not just all the practical stuff. I think the hanging out, the being friends, enjoying each other’s company gets pushed aside by everything else: buying things for the baby, cooking, cleaning, surviving.” (Participant 16, 6 months after childbirth).

Despite reduced physical intimacy, some described the emotional bond with their partner growing stronger through the shared experience of parenting. Seeing themselves as a team created a new form of closeness that was not necessarily tied to sexual connection.

### Navigating postpartum sexual health without support

Participants experienced lack of support, information, and open dialogue about sexual health in the postpartum period. The limited postpartum care stood in contrast to the attentive support received during pregnancy and childbirth. After childbirth, the focus shifted almost entirely to the baby’s well-being, of both healthcare professionals and the participants themselves, which left their own bodies and emotional health largely unattended to.

Physical and emotional difficulties were often normalised as part of becoming a parent, and viewed as something to tolerate rather than seek support for. The standard postpartum follow-up, usually scheduled six to eight weeks after childbirth, was widely perceived as too early to capture the full scope of recovery. Participants expressed a desire for more thorough and continuing care that would include physical, emotional, and sexual health, ideally through access to a multidisciplinary team, as well as guidance before and after childbirth on common sexual changes, lowered libido, and relationship needs.

When sexual health was addressed, there was often a narrow focus on contraception. This gave the impression that the main purpose of the visit was to assess readiness to resume vaginal intercourse, and less to support broader aspects of sexual well-being. Participants described the postpartum follow-up as impersonal, even mechanical, particularly when healthcare professionals asked direct questions such as “Have you had sex yet?”, sometimes simply to determine whether a pregnancy test was needed before inserting a hormonal intrauterine device. These interactions left participants feeling pressured and misunderstood.

“It was like ‘It’s been six weeks, time to start having sex again. What contraception do you want?’ And I was just sitting there thinking this isn’t even on my mind. I was exhausted, not in a state to talk about sex. Could we talk about everything else going on in my body?” (Participant 5, 22 months after childbirth).

The lack of open discussion left some participants feeling isolated. Those who had not resumed sexual activity worried that they were abnormal, while others with a strong libido early on felt unprepared and alone in that experience.

“At first, I thought something was wrong with me because I had a strong sex drive just a few weeks after giving birth. I wasn’t in pain or anything, and I felt really emotionally connected. But I hadn’t expected that. I didn’t know that was even possible.” (Participant 8, 5 months after childbirth).

As many were still adjusting to new parenthood, raising sexual health concerns with healthcare professionals felt difficult. Those who sought care for pain, vaginal dryness, or unresolved birth injuries often felt dismissed when their symptoms were simply explained as part of “normal” recovery. This left them uncertain and unsupported. Participants also described shame or reluctance to discuss libido and emotional intimacy, issues they felt were less legitimate than physical recovery. Some expressed a general distrust in the healthcare system’s attention to women’s sexual health.

“I know it’s not always easy to be taken seriously, or even get an appointment, when it comes to women’s health and sexual health. I get this little fear that if something isn’t working, I won’t be listened to. That they’ll just say, ‘Well, you’ve had two kids, that’s how it is.’” (Participant 16, 6 months after childbirth).

## Discussion

Sexual health after childbirth was characterised by an ongoing process of reorientation. Participants described adjusting to changes in their bodies, desires, identities, relationships, and priorities, while reconsidering what sexual health meant to them. This reorientation did not necessarily involve returning to pre-pregnancy sexuality. Instead, participants described different directions, including temporarily giving less priority to sexual activity, redefining intimacy, accepting bodily changes, or finding new ways of relating to themselves and their partners. Viewed through Ahmed’s queer phenomenology [22], this process of reorientation took place within social and cultural norms that made some directions more accessible than others. Heteronormative ideals were particularly prominent. Although participants described sexual health broadly, their narratives often returned to partnered vaginal intercourse as a reference point. As Ahmed [22] argues, normative lines are easier to follow because they are already socially established and reinforced.

These heteronormative ideals were also reinforced through healthcare encounters. Participants described postpartum healthcare as focusing mainly on contraception and the resumption of penetrative sex. Previous research similarly suggests that postpartum follow-up may reinforce expectations about both the type of sexual activity and the timeframe within which women should be ready to resume it [16, 26]. Similar assumptions are evident in quantitative research, where studies often focus on vaginal intercourse and use instruments that assess sexual experiences in relation to a partner [3, 27]. These instruments are typically not designed for the postpartum period, which may limit their ability to capture experiences specific to this life phase.

Participants also described a lack of open dialogue about sexual health, both in healthcare and in everyday life. This contributed to feelings of isolation and uncertainty and reflects a gap in postpartum care, where emotional and relational aspects of sexual health remain largely unaddressed [10, 28]. The understanding of sexual health encountered in healthcare did not always align with participants’ own definitions of sexual well-being. When their experiences or needs fell outside expected norms, they sometimes felt dismissed or overlooked, as also reported in previous research [15, 29]. Through Ahmed’s concept of disorientation [22], these experiences can be understood as difficulties in finding direction when established ways of understanding sexuality no longer correspond with lived experience. As participants reoriented their sexual health after childbirth, some moved away from previously held norms and developed new understandings of sexuality, intimacy, and sexual well-being.

Reorientation after childbirth involved negotiating expectations of sexuality, but participants also negotiated normative expectations of motherhood. Rather than returning to pre-pregnancy sexual patterns, some reoriented towards caregiving, with sexuality becoming less central or temporarily set aside. This may represent another normative line, in which mothers are expected to prioritise caregiving and the needs of others. Participants therefore navigated competing expectations to resume their role as a sexual partner while also embodying ideals of the self-sacrificing mother. Cultural norms can downplay women’s sexuality after childbirth while simultaneously encouraging a rapid return to pre-pregnancy sexual patterns, despite ongoing physical and emotional changes [2].

These normative expectations were also evident in how participants experienced their bodies after childbirth. A positive childbirth experience could reinforce a sense of capability and body acceptance, making it easier to be present during sexual activity. This finding is consistent with previous research linking self-confidence and body image to postpartum sexual well-being [14, 30, 31]. In contrast, participants with negative childbirth experiences sometimes felt disconnected from their bodies or perceived their bodies as having failed. As Ahmed [22] suggests, the body may become present as an object separated from the self when it does not perform as normatively expected. This interpretation aligns with previous findings that unmet expectations and social ideals surrounding motherhood and childbirth can contribute to mistrust in the body and feelings of inadequacy [32].

Bodily and sexual changes after childbirth had different meanings for participants and were not necessarily experienced as problematic. Reorientation could involve loss and grief, but also acceptance, new forms of intimacy, and greater sexual freedom. These findings challenge the assumption that changes in desire or sexual activity after childbirth necessarily indicate dysfunction. Rather, whether a change constitutes a sexual health concern depends partly on how it is experienced by the woman. This distinction highlights the importance of person-centred care that acknowledges unwanted or distressing symptoms without pathologising changes that women themselves do not perceive as problematic. Much of the literature defines sexual health through functional criteria such as desire, arousal, orgasm, and pain-free vaginal intercourse, with deviations from these criteria classified as dysfunction [33]. Reported prevalence rates of postpartum sexual dysfunction range from 45 to 83% [27, 34]. However, such classifications may pathologise changes that are common after childbirth when they are applied without considering whether women themselves experience these changes as problematic. Low desire in the early postpartum period, for example, may reflect physical recovery, fatigue, changing priorities, or a temporary reorientation away from sexual activity rather than dysfunction. Our findings support previous critiques of models that understand female sexual function as relational and visibly expressed, typically through intercourse with a male partner [3, 26, 35]. Such models may limit how sexual autonomy and identity are understood and supported after childbirth.

However, avoiding pathologisation should not result in persistent problems being normalised or overlooked. Experiences that are common during early recovery should not automatically be assumed to remain unproblematic over time. Longitudinal research shows that lack of interest in sex, vaginal dryness, and dyspareunia remain common at 6 and 12 months postpartum [34]. Persistent pain or other unwanted changes therefore warrant continued attention. The distinction should not rest solely on whether an experience falls within a predefined range of sexual function, but also on whether the woman experiences it as unwanted, distressing, or in need of support. 

Our findings have implications for postpartum care. Information about common changes in desire, intimacy, bodily comfort, and relationships could be provided both before and after childbirth, alongside reassurance that there is no expected timeframe for resuming sexual activity. Care should create opportunities for women to describe what sexual health means to them and whether changes are experienced as problematic, rather than assuming that reduced sexual activity or desire requires intervention. Conversely, symptoms or concerns that women find distressing should be acknowledged and followed up. As sexual health concerns may emerge or persist beyond the routine postpartum visit, women should have opportunities to revisit sexual health later in the postpartum period, with access to specialist or multidisciplinary support when needed.

## Strengths and limitations

A strength of this study is the diversity of the participants in terms of birth experiences, pregnancy outcomes, age, number of children, and country of birth. Digital interviews also enabled broad geographical reach across Sweden, although the lack of physical presence may have limited interpretation of non-verbal cues [36]. We acknowledge that sexual practices beyond vaginal intercourse, such as oral sex and self-pleasure, were rarely mentioned in the interviews, which possibly reflects social taboos and dominant heteronormative perspectives in discussions of sexuality during the postpartum period. Despite efforts to create a safe and open interview setting, some topics may have felt too private to share, which may have influenced how fully participants described their sexual experiences and concerns.

The sample consisted entirely of cisgender heterosexual women in predominantly heterosexual relationships, despite targeted efforts to recruit participants with diverse gender identities and sexual orientations. This limits the transferability of the findings, particularly given known disparities in sexual health among minority populations [37]. Experiences related to sexual and gender minority identities may therefore not be represented. The predominance of heterosexual perspectives may also have contributed to the strong focus on partnered vaginal intercourse in participants’ accounts. Future studies including a broader range of gender identities and sexual orientations could provide further perspectives on postpartum sexual health. In addition, the median time since childbirth was 13 months, meaning that for many participants a relatively long time had passed since their postpartum visit with the midwife. This may have influenced recall of healthcare encounters, but also means that the findings may place greater emphasis on longer-term experiences rather than those occurring in the early postpartum period [38]. The research team brought prior experience of childbirth, postpartum care, and sexual health counselling, which may have shaped the analysis. The team engaged in ongoing reflexivity and maintained openness to alternative interpretations [21].

## Conclusion

This study shows that sexual health after childbirth is a process of reorientation, shaped by bodily changes, emotional transitions, evolving roles, and cultural norms. For many participants, this involved adjusting to a new sense of self and sexuality rather than returning to pre-pregnancy sexual practices or conforming to heteronormative expectations of postpartum recovery. While some described discovering new ways of experiencing intimacy and sexual well-being, others faced challenges such as pain, fatigue, and societal expectations that restricted opportunities for sexual expression.

Postpartum care should address sexual health proactively and in an individualised way, extending beyond contraception and the resumption of intercourse to include desire, intimacy, relationships, and bodily concerns. Care should acknowledge variation in postpartum sexuality without assuming when sexual activity should resume, while ensuring appropriate assessment and follow-up of persistent symptoms or distress. Future research should evaluate approaches for integrating sexual health support into routine postpartum care and include participants with more diverse gender identities, sexual orientations, and relationship constellations, as well as co-parents’ perspectives.

## Supplementary Information

Supplementary Material 1.

Supplementary Material 2.

## Data Availability

Due to confidentiality considerations and the potential risk to participant privacy, the full interview datasets cannot be shared publicly. However, anonymised excerpts may be made available from the authors upon reasonable request.

## Abbreviations

RTA: Reflexive Thematic Analysis
SRQR: Standards for Reporting Qualitative Research
WHO: World Health Organization
