# Supplementary material for: Sexual health after childbirth: a qualitative study of women’s experiences
Source: Reprod Health. 2026 Sep 25;23:174. doi: 10.1186/s12978-026-02458-y (PMC13613751; doi:10.1186/s12978-026-02458-y)
Supplement: Supplementary file 1 [file 12978_2026_2458_MOESM1_ESM.pdf]

Project: Sexual health after pregnancy and childbirth

Semi-structured Interview Guide

Background information

- Age
- Gender identity
- Highest level of education (primary school/upper secondary/university or college)
- Marital status
- Place of residence (geographical area)
- Country of birth
- Health status (underlying conditions, medication use)
- Number of children
- Most recent childbirth (date)
- Pregnancy and birth outcome (vaginal birth/caesarean/vacuum extraction; tears; bleeding; infant outcome)
- Breastfeeding (yes/no)

Interview questions and themes

1. What does sexual health mean to you?

2. If you think about your sexual health before you became pregnant and gave birth, how has it changed?

- **Physical:** the body, body perception, and self-image
- **Psychological:** feelings, experiences, perceptions, and self-esteem
- **Social:** relationships, roles, intimacy, and isolation

3. If you have experienced any complication, pain, dysfunction, or injury after childbirth that has affected your sexual health:

- How do you experience support from healthcare, your surroundings, your partner, and your social network?
- What support or interventions would you have wished for from those around you?

4. In what ways have pregnancy and childbirth enabled your sexual health?
